# Supplementary material for: Chemical Composition and Bioactivity of Essential Oil of Ten Labiatae Species
Source: Molecules. 2020 Oct 21;25(20):4862. doi: 10.3390/molecules25204862 (PMC7587947; doi:10.3390/molecules25204862)
Supplement: Supplementary file 1 [file molecules-25-04862-s001.pdf]

Supplementary Materials

Chemical Composition and Bioactivity of Essential Oil of Ten *Labiatae* Species

Mengting Liu [1,2,†], Feiya Luo [1,2,†], Zhixing Qing [1,2], Huichao Yang [1,2], Xiubin Liu [1], Zihui Yang [1,2,*] and Jianguo Zeng [1,2,*]

[1] Hunan Key Laboratory of Traditional Chinese Veterinary Medicine, Hunan Agricultural University, Changsha 410128, China; lmt19970808@163.com (M.L.); luofeiya1688@163.com (F.L.); qingzhixing@hunau.edu.cn (Z.Q.); isyanghc@163.com (H.Y.); xiubin_liu@hunau.edu.cn (X.L.)
[2] College of Veterinary Medicine, Hunan Agricultural University, Changsha 410128, China
* Correspondence: yangzihui_2006@163.com (Z.Y.); zengjianguo@hunau.edu.cn (J.Z.); Tel.: +86-150-7312-9827 (Z.Y.); Fax: +86-731-84686560 (Z.Y.)
† These authors contributed equally to this work.

Received: 18 September 2020; Accepted: 18 October 2020; Published: 18 October 2020

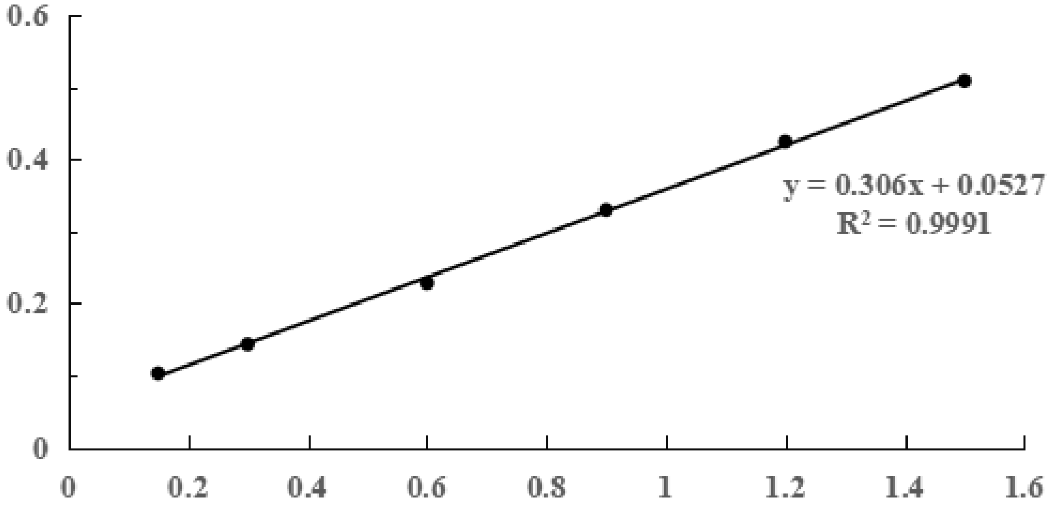

Figure S1. standard curve of FeSO

Table S1 IZD of essential oil under different pH

	S.a.			*E.coli*			*B.s.*			*S.e.*		
	pH			pH			pH			pH		
	6	7	8	6	7	8	6	7	8	6	7	8
TmEO	31.23 ±0.02	30.77 ±0.06	31.24 ±0.02	18.68 ±0.05	12.35 ±0.73	12.61 ±0.04	20.08 ±0.04	11.59 ±0.49	16.79 ±0.04	12.41 ±0.06	11.11 ±0.19	18.00 ±0.06
McEO	30.71 ±0.02	32.32 ±0.07	30.45 ±0.45	20.07 ±0.02	13.26 ±0.45	16.99 ±0.09	30.71 ±0.53	14.72 ±0.19	30.71 ±0.04	11.27 ±0.04	14.81 ±1.55	26.34 ±0.04
OvEO	32.25 ±0.02	31.82 ±0.03	32.23 ±0.04	14.81 ±0.03	10.78 ±0.49	11.08 ±0.02	17.92 ±0.05	10.52 ±0.29	15.8± 0.03	8.58± 0.04	10.71 ±0.17	18.13 ±0.14
RoEO	6.00	6.00	6.00	6.69± 0.04	6.65± 0.14	6.00	6.85± 0.02	6.46± 0.12	6.70± 0.04	6.71± 0.03	6.67± 0.21	7.84± 0.03

ObEO	21.37 ±0.11	12.98 ±0.01	20.10 ±0.03	7.06± 0.02	8.25± 0.17	6.00	7.99± 0.02	6.82± 0.31	6.59± 0.05	6.00	7.19± 0.50	6.00
MhEO	6.00	7.96± 0.34	30.31 ±0.16	6.00	8.20± 0.36	6.00	7.54± 0.49	7.75± 0.20	7.66± 0.02	6.00	7.94± 0.04	8.76± 0.02
PcEO	24.80 ±0.06	12.02 ±0.02	13.8± 0.04	6.00	6.00	6.00	6.00	6.00	6.00	7.02± 0.05	6.00	6.00
MsEO	10.47 ±1.66	9.78± 0.16	16.08 ±0.03	6.00	7.10± 0.08	6.80± 0.06	6.00	6.93± 0.07	7.14± 0.04	6.70± 0.05	7.22± 0.04	8.95± 0.02
SoEO	7.60± 0.04	7.68± 0.39	9.88± 0.05	6.00	6.00	6.00	6.00	6.00	6.00	7.22± 0.03	6.00	6.00
PfEO	15.02 ±0.12	7.81± 0.46	21.25 ±0.05	6.38± 0.01	6.00	6.00	8.52± 0.04	6.00	7.22± 0.05	8.00± 0.04	6.44± 0.04	7.77± 0.04

Table S2 The size of bacteriostatic circle of essential oil at different temperature.

	S.a.				*E.coli*				***B.s.***				***S.e.***			
	37 ℃	50 ℃	70 ℃	90 ℃	37 ℃	50 ℃	70 ℃	90 ℃	37 ℃	50 ℃	70 ℃	90 ℃	37 ℃	50 ℃	70 ℃	90 ℃
TmEO	31.24 ±0.02	32.27 ±0.04	32.76 ±0.04	32.76 ±0.04	12.35 ±0.85	11.46 ±0.02	12.10 ±0.03	11.18 ±0.02	11.59 ±0.56	19.83 ±0.04	31.30 ±0.16	20.07 ±0.03	11.11 ±0.22	20.05 ±0.02	20.90 ±0.03	20.16 ±0.03
McEO	30.45 ±0.45	31.21 ±0.06	31.22 ±0.07	31.22 ±0.07	13.26 ±0.52	16.61 ±0.03	14.86 ±0.01	18.90 ±0.03	14.72 ±0.22	30.40 ±0.21	30.43 ±0.23	30.30 ±0.16	14.81 ±1.79	30.29 ±0.11	30.34 ±0.25	30.34 ±0.13
OvEO	32.23 ±0.04	31.27 ±0.11	31.69 ±0.03	31.69 ±0.03	10.78 ±0.56	11.82 ±0.04	10.09 ±0.02	12.22 ±0.02	10.52 ±0.34	14.95 ±0.02	17.13 ±0.03	17.87 ±0.04	10.71 ±0.2	19.33 ±0.03	21.34 ±0.04	18.14 ±0.01
RoEO	7.91±0.04	8.75±0.02	9.84±0.01	9.84±0.01	6.65±0.17	6.29±0.02	6.00	6.27±0.02	6.46±0.14	6.85±0.02	6.50±0.03	7.87±0.02	6.67±0.24	6.00	8.98±0.03	8.95±0.02
ObEO	20.10 ±0.03	30.04 ±0.03	31.11 ±0.03	31.11 ±0.03	8.25±0.19	6.46±0.02	6.00	6.00	6.82±0.36	7.15±0.02	7.14±0.02	7.20±0.03	7.19±0.57	7.14±0.01	9.07±0.02	8.94±0.01
MhEO	32.12 ±0.15	32.85 ±0.13	33.13 ±0.02	33.13 ±0.02	8.2±0.41	6.00	6.00	6.00	7.75±0.23	6.49±0.03	6.00	7.04±0.01	7.94±0.05	8.56±0.02	8.55±0.02	8.46±0.03
PcEO	17.51 ±0.04	20.25 ±0.02	21.93 ±0.03	21.93 ±0.03	6.00	6.00	6.00	6.00	6.00	6.00	6.00	6.00	6.00	6.00	6.00	6.00
MsEO	33.20 ±0.05	33.04 ±0.05	33.55 ±0.01	33.55 ±0.01	7.1±0.1	6.85±0.02	6.00	6.45±0.02	6.93±0.08	7.44±0.01	8.05±0.02	8.33±0.02	7.22±0.05	9.39±0.02	11.12 ±0.02	11.05 ±0.02
SoEO	16.40 ±0.03	14.33 ±0.03	15.88 ±0.03	15.88 ±0.03	6.00	6.00	6.00	6.00	6.00	6.00	6.00	6.00	6.00	6.00	6.00	6.00
PfEO	21.25 ±0.04	17.51 ±0.04	21.32 ±0.04	21.32 ±0.04	6.00	6.00	6.00	6.00	6.00	6.00	6.00	6.00	6.44±0.04	9.17±0.02	14.30 ±0.03	14.08 ±0.02

Table S3 IZD of essential oil under different UV irradiation time

	S.a.				*E.coli*				*B.s.*				*S.e.*			
	duration(min)				duration(min)				duration(min)				duration(min)			
	0	10	20	30	0	10	20	30	0	10	20	30	0	10	20	30
TmEO	30.77 ±0.07	31.46 ±0.39	31.45 ±0.29	32.26 ±0.05	12.35 ±0.85	12.27 ±0.03	16.98 ±0.03	11.13 ±0.02	12.35 ±0.85	16.69 ±0.03	17.44 ±0.04	25.67 ±0.02	12.35 ±0.85	22.06 ±0.02	21.45 ±0.02	21.54 ±0.01
McEO	32.32 ±0.08	32.54 ±0.16	32.43 ±0.21	31.26 ±0.14	13.26 ±0.52	15.14 ±0.04	21.09 ±0.03	18.33 ±0.03	13.26 ±0.52	24.83 ±0.03	30.31 ±0.16	31.31 ±0.05	13.26 ±0.52	31.84 ±0.05	32.40 ±0.16	24.84 ±0.01
OvEO	31.82 ±0.03	32.66 ±0.53	33.03 ±0.47	30.34 ±0.12	10.78 ±0.56	11.34 ±0.02	15.06 ±0.05	10.06 ±0.01	10.78 ±0.56	15.84 ±0.04	18.67 ±0.04	19.45 ±0.02	10.78 ±0.56	18.14 ±0.01	18.86 ±0.01	17.56 ±0.01
RoEO	6±0	9.94± 0.04	8.86± 0.01	7.26± 0.02	6.65± 0.17	6.34± 0.04	6.56± 0.04	6.00	6.65± 0.17	7.55± 0.02	6.94± 0.02	7.58± 0.01	6.65± 0.17	8.56± 0.02	8.66± 0.01	6.65± 0.02
ObEO	12.98 ±0.02	21.58 ±0.04	21.24 ±0.02	19.72 ±0.03	8.25± 0.19	6.00	7.07± 0.04	6.55± 0.02	8.25± 0.19	6.95± 0.02	8.14± 0.01	7.84± 0.01	8.25± 0.19	8.23± 0.03	8.05± 0.02	6.96± 0.01
MhEO	7.96± 0.39	27.32 ±0.03	31.49 ±0.25	31.13 ±0.07	8.2±0. 41	6.00	6.00	6.00	8.2±0. 41	8.26± 0.01	7.50± 0.03	6.83± 0.02	8.2±0. 41	8.23± 0.02	9.55± 0.02	8.54± 0.01
PcEO	12.02 ±0.03	16.83 ±0.03	15.05 ±0.02	15.96 ±0.01	6.00	6.00	6.00	6.00	6.00	6.00	6.00	6.00	6.00	6.00	6.00	6.00
MsEO	9.78± 0.18	26.18 ±0.04	18.83 ±0.04	25.85 ±0.02	7.1±0. 1	7.09± 0.03	6.96± 0.02	6.64± 0.01	7.1±0. 1	7.85± 0.02	7.79± 0.02	7.14± 0.01	7.1±0. 1	9.94± 0.01	12.25 ±0.02	7.96± 0.01
SoEO	7.68± 0.45	7.88± 0.05	9.96± 0.01	9.14± 0.02	6.00	6.00	6.00	6.00	6.00	6.00	6.00	6.00	6.00	6.84± 0.01	6.96± 0.01	6.00
PfEO	7.81± 0.53	22.84 ±0.05	24.70 ±0.05	18.34 ±0.03	6.00	6.00	6.00	6.00	6.00	8.03± 0.03	8.36± 0.02	8.39± 0.02	6.00	10.06 ±0.02	11.13 ±0.02	8.94± 0.01

Table S4 Diverse cycles for the analyses of essential oils of 10 plants.

	It	Rt	Tp	Rt	Tp	Rt	Tp	Rt	Tp	Rt	Dt
TmEO	40 ℃	2 min	3 ℃/min,130 ℃	1 min	5 ℃/min,240 ℃	2 min	-	-			57 min
McEO	40 ℃	2 min	2 ℃/min,120 ℃	1 min	5 ℃/min,200 ℃	2 min	-	-			61 min
OvEO	60 ℃	2 min	3 ℃/min,100 ℃	2 min	1 ℃/min,110 ℃	1 min	3 ℃/min,190 ℃	2 min			57 min
RoEO	40 ℃	2 min	3 ℃/min,100 ℃	1 min	2 ℃/min,110 ℃	1 min	4 ℃/min,220 ℃	2 min			58.5 min
ObEO	40 ℃	2 min	2 ℃/min,100 ℃	1 min	0.5 ℃/min,108 ℃	1 min	6 ℃/min,150 ℃	1 min	3 ℃/min,240 ℃	2 min	90 min
MhEO	40 ℃	2 min	2 ℃/min,110 ℃	1 min	4 ℃/min,200 ℃	2 min					62.5 min
PcEO	60 ℃	1 min	10 ℃/min,100 ℃	1 min	2 ℃/min,150 ℃	1 min	1 ℃/min,160 ℃	1 min	4 ℃/min,220 ℃	2 min	60 min
MsEO	40 ℃	2 min	2 ℃/min,110 ℃	1 min	4 ℃/min,200 ℃	2 min					62.5 min
SoEO	40 ℃	2 min	3 ℃/min,130 ℃	1 min	5 ℃/min,240 ℃	2 min					57 min
PfEO	60 ℃	2 min	5 ℃/min,100 ℃	1 min	2 ℃/min,160 ℃	2 min	4 ℃/min,200 ℃	2 min			55 min

It: Initial temperature; Rt: Retention time; Tp: Temperature-raising program; Dt: detection time
